# Retraction Note: Anti-hyperalgesic properties of a flavanone derivative Poncirin in acute and chronic inflammatory pain models in mice

**DOI:** 10.1186/s40360-023-00679-6

**Published:** 2023-06-30

**Authors:** Ruqayya Afridi, Ashraf Ullah Khan, Sidra Khalid, Bushra Shal, Hina Rasheed, Muhammad Zia Ullah, Omer Shehzad, Yeong Shik Kim, Salman Khan

**Affiliations:** 1 Department of Pharmacy, Faculty of Biological Sciences, Quaid-I-Azam University, Islamabad, Pakistan; 2 Department of Pharmacy, Abdul Wali Khan University, Mardan, Pakistan; 3 College of Pharmacy, Seoul National University, Seoul, 151-742 South Korea

**Retraction Note: BMC Pharmacol Toxicol 20, 57 (2019)**

**https://doi.org/10.1186/s40360-019-0335-5**

The Editor has retracted this article. After publication, concerns were raised regarding high similarity between the images presented in Fig. 11 of this article and Fig. 6 in the authors' earlier work [1]. The authors have stated that the same control groups were used in both articles; however, the animal descriptions and ethics approval numbers are different in the two publications. The authors have also provided raw data to address these concerns, but some of the data do not match the published figures. The Editor therefore no longer has confidence in the presented data.

None of the authors have responded to correspondence from the Editor about this retraction notice.
